# Prevalence of and factors associated with herpes zoster in England: a cross-sectional analysis of the Health Survey for England

**DOI:** 10.1186/s12879-022-07479-z

**Published:** 2022-06-01

**Authors:** Sharon L. Cadogan, Jennifer S. Mindell, Judith Breuer, Andrew Hayward, Charlotte Warren-Gash

**Affiliations:** 1 Department of Non-Communicable Disease Epidemiology, London School of Hygiene and Tropical and Medicine, London, UK; 2 Research Department of Epidemiology and Public Health, University College London, London, UK; 3 Division of Infection and Immunity, University College London, London, UK; 4 Institute of Epidemiology and Health Care, University College London, London, UK

**Keywords:** Herpes zoster, Shingles, Risk factor, Prevalence

## Abstract

**Background:**

Herpes zoster (commonly called shingles) is caused by the reactivation of varicella zoster virus, and results in substantial morbidity. While the risk of zoster increases significantly with age and immunosuppression, relatively little is known about other risk factors for zoster. Moreover, much evidence to date stems from electronic healthcare or administrative data. Hence, the aim of this study was to explore potential risk factors for herpes zoster using survey data from a nationally-representative sample of the general community-dwelling population in England.

**Methods:**

Data were extracted from the 2015 Health Survey for England, an annual cross-sectional representative survey of households in England. The lifetime prevalence of self-reported herpes zoster was described by age, gender and other socio-demographic factors, health behaviours (physical activity levels, body mass index, smoking status and alcohol consumption) and clinical conditions, including; diabetes, respiratory, digestive and genito-urinary system and mental health disorders. Logistic regression models were then used to identify possible factors associated with shingles, and results were presented as odds ratios with 95% confidence intervals.

**Results:**

The lifetime prevalence of shingles among the sample was 11.5% (12.6% among women, 10.3% among men), which increased with age. After adjusting for a range of covariates, increased age, female gender (odds ratio: 1.21; 95%CI: 1.03, 1.43), White ethnic backgrounds (odds ratio: 2.00; 95%CI: 1.40, 2.88), moderate physical activity 7 days per week (odds ratio: 1.29; 95%CI: 1.01, 1.66) and digestive disorders (odds ratio: 1.51; 95%CI: 1.13, 1.51) were each associated with increased odds of having had herpes zoster.

**Conclusions:**

Age, gender, ethnicity and digestive disorders may be risk factors for herpes zoster among a nationally representative sample of adults in England. These potential risk factors and possible mechanisms should be further explored using longitudinal studies.

**Supplementary Information:**

The online version contains supplementary material available at 10.1186/s12879-022-07479-z.

## Background

Primary infection by varicella zoster virus (VZV) typically results in childhood chickenpox, after which the virus establishes latency in the peripheral ganglia. Herpes Zoster (HZ), more commonly known as shingles, occurs when the dormant VZV reactivates, usually decades later, following reduced cell-mediated immunity [1]. The incidence and severity of HZ increases markedly after 50 years of age [2–6]. For example, in the United Kingdom (UK), incidence of HZ rises from 7.1 per 1000 person years among 60- to 64-year-olds to 12.2 per 1000 person years among those over 85 [7]. HZ typically presents as a painful, erythematous, maculopapular rash, usually lasting 2–4 weeks [8]. Although antiviral therapy is an effective treatment for HZ, to reduce the duration and severity of the illness, many vascular, visceral, and neurological complications of HZ exist [9]. One major complication associated with HZ is postherpetic neuralgia (persistent pain for over 90 days after shingles onset), which occurs in approximately 20% of HZ patients, particularly older patients [10].

In addition, the incidence of HZ appears to be rising [11]. However, the causes of VZV reactivation and HZ are not completely known. Some risk factors, such as older age and immunosuppression, are well established [5, 12], but they cannot explain fully the epidemiology of HZ [13]. Other potential risk factors from the literature include female gender [14], ethnicity [15, 16], family history of HZ [17], and chronic health conditions [18], such as inflammatory bowel disease [19], diabetes [20] and chronic obstructive pulmonary disorder. However, findings have been inconsistent. Moreover, much of the literature on HZ comes from electronic health records or other administrative data used for healthcare billing purposes [3, 4, 18, 21, 22], which may under-estimate the prevalence of HZ in the population, especially for mild cases and those affecting younger people. Very limited evidence exists from population surveys [16, 23] using self-reported HZ, which has previously demonstrated accuracy and suitability for epidemiological research [24]. In addition, other potential risk factors, such as lifestyle factors, may be poorly recorded in electronic health records and may be better explored using survey data.

Hence, this study aimed to describe the lifetime prevalence of self-reported HZ by age and gender and investigate other factors associated with self-reported HZ, using the Health Survey for England (HSE), 2015. This collected detailed information on mental and physical health, health-related behaviours, and objective physical and biological measures, in relation to demographic and socio-economic characteristics.

## Methods

### Health Survey for England

Briefly, the HSE is an annual cross-sectional survey of a new, nationally-representative sample of the English population, selected anew each year. It is a multistage, stratified, random probability sample of private households in England and is used to estimate prevalence of health conditions and disease risk factors, as well as to plan health services and monitor government performance against policy targets. The survey had a response rate of 57% for adults overall (85% of adults in responding households) [25]. The methodology of the HSE has been published elsewhere [25]. In brief, each survey contains a range of health and socio-demographic related questions as well as measurements taken by a trained interviewer (height, weight) and, at a follow-up visit for consenting participants, a nurse (including waist circumference, blood pressure, and urine, blood and/or saliva samples) [24, 25]. In addition, each year the survey includes different modules focusing on a specific topic: in 2015, HZ was included in the core questionnaire [26].

### Study population

The sample comprised 8022 adults (aged 16 years or over), participating in HSE 2015. Data collection was conducted in participants’ homes via face-to-face Computer Assisted Personal Interview and self-completion methods and is explained fully elsewhere [25].

### Herpes zoster

The outcome of interest for this study was self-reported shingles (including both non-diagnosed and doctor-diagnosed shingles). This was defined by a positive response to the following question: *“Shingles is a painful blistering rash caused by the same virus that causes chickenpox. Have you ever had shingles?”* If participants responded yes, they were also asked when they had shingles using the following two questions: *“What year did you have shingles?*” and *“What age were you when you had shingles?”.*

### Health behaviours

Health behaviours included self-reported physical activity levels (how many days over the past seven days participants performed moderate physical activity); smoking status (non-smoker, ex-regular/ex-occasional smoker, or current cigarette smoker); alcohol consumption (how many units of alcohol consumed on the heaviest drinking day in the past seven days); and Body Mass Index (BMI, calculated using measured height and weight where available and supplemented with self-reported BMI to avoid dropping participants with no height and/or weight measurements). Participants were classified into one of three categories of BMI: < 25 kg/m^2^, not overweight or obese; 25 to < 30 kg/m^2^, overweight; and 30 kg/m^2^ or more, obese.

### Socio-demographic characteristics

Data on gender (male or female), age (in 10-year categories), household size, and ethnicity were collected in the face-to-face interview. For the purpose of this research, ethnicity was recoded to White or Non-White due to small numbers in each minority ethnic group.

Area-level deprivation was based on the participant’s address and defined using the Index of Multiple Deprivation (IMD) 2015, expressed in quintiles. The lowest quintile indicates the lowest levels of deprivation (IMD 0.37–8.32); the highest quintile indicates that the neighbourhood experienced the highest levels of deprivation (34.42–85.46).

Participants’ wellbeing was defined using the Warwick-Edinburgh Mental Well-Being Scale (WEMWBS), which was self-completed confidentially as part of the interviewer visit. WEMWBS scores were categorised into three groups: more than one SD from the mean in either direction (top 15 centiles: WEMWBS score 60–70; and bottom 15 centiles: WEMWBS score 14–42) and the remainder (16th percentile to 84th percentile: WEMWBS score 43–59).

### Clinical conditions

Health conditions included respiratory system disorders, digestive disorders, genito-urinary system disorders and mental health disorders. Data for these four conditions were derived from responses to the following question on long-lasting illness: *Do you have any physical or mental health conditions or illnesses lasting or expecting to last 12 months or more?* Answers to the follow-up question to those responding positively, asking what the condition was, were coded to ICD-10 chapters. We also included doctor-diagnosed diabetes, elicited from a direct question (*Have you been told by a doctor or nurse that you had diabetes?).*

### Statistical analysis

All analyses were performed using Stata MP, version 16.0 (Stata Corp LP, College Station, TX, USA). Probability weights were applied to the data using weighting data supplied with the dataset to account for the complex survey sampling strategy, including probability of address selection (smaller regions were oversampled), dwelling unit selection (only one dwelling unit selected per address) and household selection (only one household selected per dwelling unit); household non-response; and individual non-response to interview [26]. These weights corrected the distribution of household members to match population estimates for age/sex groups and region [26]. Further details on the weighting process can be found elsewhere [26].

The lifetime prevalence of self-reported HZ was described by age, gender, and study sample characteristics, and compared using the chi-squared test. Factors to analyse for potential associations with HZ (exposure variables) were selected based on previous literature. Logistic regression models (adjusted for age and gender) were used to estimate the strength of the association of each exposure with self-reported HZ. Multivariate regression models were then used to compute adjusted odds ratios, accounting for potential confounders. The standard errors of the key effect estimates in the ‘fully adjusted’ model and the corresponding standard errors in the initial models (adjusted only for age and gender) were compared to check for multicollinearity. Potential effect modifiers (age and gender) were evaluated by adding interaction terms into the multivariate model. Results are reported as adjusted odds ratios (aOR), with their corresponding 95% confidence intervals (CIs) and p-values (level of significance: < 0.05).

### Ethical approval

This study was approved by the London School of Hygiene and Tropical Medicine Ethics Committee (Ref: 17836). Ethical approval for the 2015 Health Survey for England was obtained from the West London Research Ethics Committee (Ref: 14/LO/0862) and informed consent obtained from each participant. All methods were carried out in accordance with relevant guidelines and regulations.

## Results

Among 8034 adults (aged 16 or over) who participated in the 2015 survey, 8022 (99.9%) responded to additional questions about shingles. A description of participants (unweighted data) is shown in Additional file 1: Table S1. The median age of the overall study population was 51 (IQR: 36–66). Among those who reported previously having shingles, the median age was 63 (IQR: 49–74), compared to 49 years (IQR: 35–64) among participants who never had shingles. Among those, who reported previously having shingles, the median age of shingles onset was 42 (IQR: 25–57). Reporting a history of shingles was more common among women, individuals of older age, those of White ethnicity and those with certain medical conditions, including diabetes respiratory and digestive disorders.

### Lifetime prevalence of shingles

The overall lifetime prevalence of shingles was 11.5%, (12.6% among women and 10.3% among men). Table 1 presents the lifetime prevalence of shingles, by sample characteristics. The lifetime prevalence of shingles was higher among those of older change, White ethnicity, smaller household size and ex-smokers.

**Table 1:** Lifetime prevalence of shingles, by sample characteristics

	Shingles % (95% CI)	p-value^
Gender		
Male	10.3 (9.3–11.5)	0.002
Female	12.6 (11.6–13.7)	
Age-group		
16–24	3.9 (2.6–5.9)	0.000
25–34	5.5 (4.2–7.1)	
35–44	7.6 (6.1–9.4)	
45–54	11.2 (9.3–13.3)	
55–64	14.9 (12.97–17.2)	
65–74	19.1 (17.0–21.5)	
75 +	25.9 (23.2–28.7)	
Ethnicity		
Non-White	3.8 (2.8–5.2)	0.000
White	12.6 (11.8–13.5)	
Missing	8.2 (1.9–29.2)	
Well-being (WEMWBS score)		
Bottom 15th centile	12.5 (10.6–12.9)	0.584
Remainder	11.4 (10.4–12.4)	
Top 15th centile	12.1 (10.2–14.1)	
Missing	10.5 (8.6–12.9)	
Household size		
1	16.1 (14.3–18.1)	0.000
2	14.1 (12.8–15.5)	
3	9.1 (7.4–11.1)	
4	8.2 (6.6–10.1)	
5 or more	6.5 (4.5–9.3)	
BMI^c,d^		
Not overweight or obese	10.4 (9.3–11.7)	0.143
Overweight	12.3 (11.1–13.7)	
Obese	11.8 (10.4–13.4)	
Missing	13.4 (8.4–20.6)	
Area level deprivation quintiles^a^		
Least deprived	12.4 (10.9–14.0)	0.001
Second lowest	13.1 (11.1–15.6)	
Middle	11.8 (10.2–13.7)	
Second highest	11.8 (10.2–13.7)	
Most deprived	8.3 (7.0–9.0)	
Physical activity (moderate)^e^		
No days	11.5 (10.2–12.9)	0.006
1–2 days	9.4 (8.1–10.8)	
3–4 days	11.8 (9.9–14.0)	
5–6 days	11.5 (8.9–14.7)	
7 days	14.2 (12.2–16.5)	
Smoking status		
Never smoker	10.1 (9.1–11.2)	0.000
Ex-regular/Ex-occasional	14.9 (13.5–16.4)	
Current	10.3 (8.6–12.2)	
Missing	1.9 (0.5–7.5)	
Alcohol consumption^b^		
None	10.3 (9.2–11.4)	0.000
≤ 2 units	14.4 (12.6–16.4)	
> 2 and ≤ 4 units	10.7 (8.9–12.8)	
> 4 and ≤ 8 units	12.9 (10.8–15.2)	
8 + units	10.4 (8.5–12.7)	
Missing	23.6 (3.6–72.0)	
Clinical conditions		
Diabetes	15.1 (12.4–18.2)	0.015
Respiratory Disease	17.0 (14.0–20.5)	0.000
Digestive disorders	19.3 (15.7–23.5)	0.000
Genito-urinary disorders	20.3 (15.1–26.8)	0.000
Mental health condition	12.2 (9.3–15.8)	0.438

*BMI* body mass index, *WEMWBS* Warwick-Edinburgh Mental Well-being Scale

^a^Area level deprivation defined using Index of Multiple Deprivation 2015 scores

^b^Units of alcohol consumed on heaviest drinking day in past 7 days

^c^BMI was calculated using measured height and weight where available and supplemented with self-reported BMI among participants with no height and/or weight measurements (BMI categories: < 25 kg/m^2^, not overweight or obese; 25 to < 30 kg/m^2^, overweight; and 30 kg/m^2^ or more, obese)

^d^Underweight included with normal weight due to small numbers

^e^Number of days performing moderate physical activity in the past 7 days

^χ^2^ tests were used to determine the statistical significance of any difference in the distributions. Statistical significance level: p value < 0.05

### Risk factors for shingles

Table 2 presents the findings of the ‘age and sex’ and ‘fully adjusted’ multivariate logistic regression models. After adjusting for a range of socio-economic and clinical risk factors, age, gender, ethnicity and performing moderate physical activity 7 days per week were each found to be associated with HZ. Age was a strong predictor of HZ risk, with significantly increased odds among older age groups. The odds of having had HZ was also 21% higher in females, compared with males (aOR: 1.21; 95%CI: 1.03, 1.43), and people from White ethnic backgrounds had twice the odds of having had shingles, compared with those of Non-White ethnicity (aOR: 2.00; 95%CI: 1.40, 2.88). People who reported performing moderate physical activity seven days per week, compared to none, also had higher odds of reporting HZ (aOR 1.29; 95%CI: 1.01, 1.66).

**Table 2:** Factors associated with reporting HZ—findings from age and sex adjusted and fully adjusted multivariate logistic regression model

Variable	Age and sex adjusted		Fully-adjusted	
	OR (95% CI)	p-value	aOR (95% CI)	p-value
Gender				
Male	Ref.		Ref.	
Female	1.21 (1.05–1.40)	0.010	1.21 (1.03–1.43)	0.022
Age-group				
16–24	Ref.		Ref.	
25–34	1.41 (0.86–2.24)	0.175	1.32 (0.77–2.25)	0.311
35–44	1.99 (1.24–3.21)	0.005	1.97 (1.19–3.28)	0.009
45–54	3.06 (1.96–4.78)	0.000	2.83 (1.74–4.61)	0.000
55–64	4.28 (2.77–8.98)	0.000	3.72 (2.32–5.97)	0.000
65–74	5.75 (3.69–8.98)	0.000	5.26 (3.24–8.54)	0.000
75 +	8.41 (5.41–13.10)	0.000	7.76 (4.73–12.76)	0.000
Ethnicity				
Non-White	Ref.		Ref.	
White	2.67 (1.92–3.70)	0.000	2.00 (1.40–2.88)	0.000
Well-being (WEMBMS score)				
Bottom 15th centile	Ref.		Ref.	
Remainder	0.92 (0.72–1.17)	0.487	0.95 (0.74–1.22)	0.674
Top 15th centile	0.88 (0.66–1.17)	0.391	0.98 (0.73–1.34)	0.922
Household size				
1	Ref.			
2	1.03 (0.86–1.24)	0.750	1.06 (0.87–1.31)	0.552
3	1.04 (0.79–1.35)	0.790	1.11 (0.81–1.51)	0.525
4	1.06 (0.76–1.43)	0.710	1.18 (0.84–1.65)	0.333
5 or more	0.89 (0.57–1.37)	0.586	0.97 (0.60–1.59)	0.915
BMI^a,b^				
Not overweight or obese	Ref.		Ref.	
Overweight	0.97 (0.82–1.16)	0.743	0.95 (0.78–1.15)	0.581
Obese	0.91 (0.75–1.11)	0.353	0.88 (0.70–1.09)	0.243
Area level Deprivation^c^				
Least deprived	Ref.		Ref.	
Second-lowest	1.15 (0.90–1.46)	0.267	1.13 (0.87–1.47)	0.349
Middle	1.03 (0.82–1.29)	0.805	1.06 (0.83–1.34)	0.649
Second-highest	1.12 (0.90–1.40)	0.300	1.12 (0.87–1.44)	0.385
Most deprived	0.82 (0.65–1.04)	0.108	0.91 (0.69–1.19)	0.482
Physical activity—moderate				
No days	Ref.		Ref.	
1–2 days	0.92 (0.75–1.13)	0.423	0.86 (0.68–1.07)	0.179
3–4 days	1.23 (0.97–1.56)	0.088	1.10 (0.86–1.42)	0.449
5–6 days	1.21 (0.88–1.66)	0.234	1.11 (0.79–1.54)	0.544
7 days	1.40 (1.12–1.74)	0.003	1.29 (1.01–1.66)	0.043
Smoking status				
Never smoker	Ref.		Ref.	
Ex regular/Ex occasional smoker	1.21 (1.02–1.43)	0.026	1.09 (0.91–1.30)	0.362
Current smoker	1.24 (0.99–1.57)	0.066	1.22 (0.94–1.57)	0.132
Alcohol consumption^d^				
None	Ref.		Ref.	
≤ 2 units	1.27 (1.04–1.55)	0.020	1.15 (0.92–1.43)	0.218
> 2 and ≤ 4 units	1.01 (0.80–1.27)	0.961	0.98 (0.76–1.27)	0.882
> 4 and ≤ 8 units	1.40 (1.12–1.76)	0.004	1.17 (0.91–1.51)	0.224
8 + units	1.34 (1.03–1.75)	0.027	1.22 (0.91–1.63)	0.177
Clinical conditions				
Diabetes^e^	1.13 (0.89–1.44)	0.300	1.00 (0.76–1.33)	0.977
Respiratory disease	1.47 (1.14–1.90)	0.003	1.31 (0.96–1.77)	0.085
Digestive disorders	1.48 (1.15–1.92)	0.003	1.51 (1.13–2.01)	0.005
Genito urinary disorders	1.40 (0.95–2.05)	0.087	1.25 (0.81–1.94)	0.317
Mental health condition	1.26 (0.91–1.75)	0.165	1.15 (0.80–1.65)	0.448

*BMI* body mass index, *WEMWBS* Warwick-Edinburgh Mental Well-being Scale

^a^BMI was calculated using measured height and weight where available and supplemented with self-reported BMI among participants with no height and/or weight measurements (BMI categories: < 25 kg/m^2^, not overweight or obese; 25 to < 30 kg/m^2^, overweight; and 30 kg/m^2^ or more, obese)

^b^Underweight included with normal weight due to small numbers

^c^Area level deprivation defined using Index of Multiple Deprivation 2015 scores

^d^Units of alcohol consumed on heaviest drinking day in past 7 days

^e^Doctor-diagnosed diabetes

^Statistical significance level: p value < 0.05

The risk of HZ was also increased by 51% in participants who reported having digestive disorders (aOR: 1.51; 95%CI: 1.13, 2.01). No other clinical, lifestyle or sociodemographic risk factors were found to be associated with herpes zoster (see Table 2).

We also explored how the effect of possible risk factors varied by gender and age. We found some evidence that the odds of self-reported HZ varied by gender for ethnicity, smoking status and digestive disorders. Additional file 1: Table S2 presents the results from the fully adjusted model; stratified by gender.

## Discussion

Using nationally-representative survey data to explore the prevalence of and potential risk factors for HZ in England, this study found the overall weighted lifetime prevalence of shingles to be 11.5%. After adjusting for a range of factors, increased age, female gender, White ethnicity, performing moderate physical activity 7 days per week and digestive disorders were associated with increased odds of HZ.

### Demographic and lifestyle factors and HZ

In this study, older age was significantly associated with increased odds of HZ. Age is the most well-established risk factor for HZ in the literature [13, 17, 27, 28]. In a recent meta-analysis, pooled analysis of 39 studies examining age as a potential risk factor found older age was associated with a significant increase in HZ risk (RR = 1.65, 95% CI, 1.37–1.97) [28]. The elevated risk in the older patients is likely explained by the fact that the immune system progressively deteriorates as individuals age.

We also found women had higher odds of HZ, even after adjusting for age, which is consistent with previous research [14, 28]. A review exploring gender differences in the incidence of HZ proposed that gender biases during diagnosis may be important [29]. For example, women may be more likely to attend their GP with shingles symptoms. It is also possible that hormonal or biological differences between genders could play a role. For example, the menopause transition period females go through is also suspected to be responsible for increased female HZ incidence due to hormonal changes to their immune response.

Consistent with previous research [15, 17, 21, 27, 28], people from White ethnic backgrounds were also found to have twice the odds of HZ, compared with those from Non-White ethnic backgrounds. Possible reasons for the lower risk of zoster among different racial/ethnic groups may be explained by differences in household composition; black individuals may have elevated exposure to varicella throughout their lifetime, which boosts immune response to VZV [13, 30]. It has also been suggested that this reduced risk may result from maintenance of VZV-specific immunity in older age due to the protective effect associated with ethnicity among people born in countries with evidence of late-onset of varicella (e.g., Central America) [13, 31].

We also found that performing moderate physical activity seven days per week increased the risk of HZ by 29% compared to those who did not exercise. These findings are not consistent with previous epidemiological studies examining physical activity as a possible risk factor for HZ, which found no association [32, 33]. However, research suggests that vigorous physical activity may be immunosuppressive [34], and hence, could potentially play a role in the reactivation of varicella zoster through this mechanism. This could possibly explain our findings; however, it is also important to note that we used self-reported physical activity data, which may be susceptible to recall or misclassification bias.

Other demographic and lifestyle factors, including deprivation, BMI, smoking and alcohol consumption were found to have no association with HZ. This is consistent with previous research examining the role of lifestyle and anthropometric factors and risk of zoster [33].

### Chronic clinical conditions and HZ

In this current study, digestive disorders were associated with higher odds of HZ. This association may be related to immunosuppression, which is a well-established risk factor for HZ [12, 21]. For example, patients with inflammatory bowel disease routinely use immunosuppressive medications including corticosteroids and calcineurin inhibitors. Similarly, respiratory conditions have also previously been linked to increased risk for HZ [13, 21]. We found an association between respiratory disorders and HZ in our age- and gender-adjusted analyses (p < 0.001); however, this association lost significance once adjusted for other potential confounders. It has been suggested that the link between respiratory conditions and HZ may also be explained by routine use of immunosuppressants, such as inhaled or systemic corticosteroids used for management of chronic obstructive pulmonary disorder [35]—although we were unable to assess the role of immunosuppression directly in our study.

Other chronic conditions included in this study were not found to be associated with HZ. For example, this study found no association between doctor-diagnosed diabetes and the odds of HZ. This is in contrast to many previous studies which have linked diabetes to higher odds of HZ [20, 27, 36]. However, the strength of the association between these studies varied, depending on methodological aspects such as age adjustments or comorbidities. Moreover, similar to our findings, another UK study using electronic health record data found no association between HZ and diabetes overall (adjusted odds ratio 1.02, 0.99 to 1.05), although it did report an association between type 1 diabetes and HZ [21]. Our findings may also be explained by undiagnosed diabetes, which some studies have suggested is high among HZ patients [37], suggesting that routine screening for diabetes could be beneficial in HZ patients.

Finally, we found no association between mental health conditions and HZ. Previous literature have linked self-reported depression with HZ [16, 17], however, the strength of the association varied greatly, and could be due to reverse causality, as people with postherpetic neuralgia may develop depressive symptoms as a result of the chronic pain [38].

### Strengths and limitations

This study has several strengths. First, it utilises data from a nationally representative survey, containing detailed information on socio-demographic and lifestyle factors, which may not be available in health records. The survey also provides self-reported data on HZ which is more sensitive than relying on information from medical records and has been shown to be valid in some contexts.

However, some limitations also exist. The cross-sectional design of this study may not provide an accurate picture of health at diagnosis, in particular for clinical conditions. i.e., cannot assess temporality (resulting in potential for reverse causality). Further, because we used survey data to measure lifestyle risk factors, non-response and misclassification of risk factors may exist. Longitudinal studies could be utilised to measure detailed lifestyle factors such as physical activity and follow up for incident HZ diagnoses. Data collected on clinical risk factors and medications (in particular immunosuppressive conditions and therapies) was also limited, compared with other data sources, such as electronic health records. Also, we did not have information on HZ vaccine, however this was introduced in the UK in 2013 for a very limited subset of people aged 70 years, with a catch-up campaign for those aged 79, so is unlikely to have affected results in younger adults. In addition, as with all observational designs, some possible unmeasured bias is a limitation. HZ is primarily diagnosed clinically; using a self-reported outcome may lead to either over-estimation of prevalence, e.g., due to inaccurate identification of other rashes as shingles, or under-estimation, perhaps due to unwillingness to report infection. However, self-reported HZ has previously been shown to be accurate [24] compared with physician diagnosis of HZ and with a HZ verification questionnaire. In that study, the agreement of self-reports to physician diagnosis was 98.9%; the sensitivity and specificity were 100% and 98.4%, respectively [22]. Moreover, HZ has a highly characteristic clinical presentation and self-report data generally stems from a clinical diagnosis by a general practitioner or other healthcare professional [23].

## Conclusions

Using a nationally-representative general population sample, we have confirmed the higher prevalence in older people and women of having had herpes zoster. Our data also showed an association with White ethnicity and with reported digestive disorders. These potential risk factors should be explored in future longitudinal studies for confirmation and to investigate possible mechanisms.

## Supplementary Information

**Additional file 1: Table S1.** Unweighted descriptive characteristics of participants, by shingles status. **Table S2.** Factors associated with reporting HZ, by gender (fully adjusted model).

## Data Availability

The data used in the study was under license from the Health Survey for England. As a result, data are not publicly available, but can be requested from the HSE.

## Abbreviations

aOR: Adjusted odds ratio
BMI: Body mass index
CI: Confidence interval
HSE: Health Survey for England
HZ: Herpes zoster
OR: Odds ratio
VZV: Varicella zoster virus
