# Supplementary material for: Prevalence of and factors associated with herpes zoster in England: a cross-sectional analysis of the Health Survey for England
Source: BMC Infect Dis. 2022 Jun 1;22:513. doi: 10.1186/s12879-022-07479-z (PMC9158364; doi:10.1186/s12879-022-07479-z)
Supplement: Supplementary file 1 — Additional file 1: Table S1. Unweighted descriptive characteristics of participants, by shingles status. Table S2. Factors associated with reporting HZ, by gender (fully adjusted model). [file 12879_2022_7479_MOESM1_ESM.docx]

**Table S1** Unweighted descriptive characteristics of participants, by shingles status

|  | **Overall**  **N (%)** | **Shingles**  **N (%)** | **No shingles**  **N (%)** | **P-value^** |
| --- | --- | --- | --- | --- |
|  | 8,022 (100.0) | 1,012 (12.6) | 7,010 (87.4) |  |
| **Gender** |  |  |  | 0.004 |
| Male | 3,568 (44.5) | 407 (40.2) | 3,161 (45.1) |  |
| Female | 4,454 (55.5) | 605 (59.8) | 3,849 (54.9) |  |
| **Age-group** |  |  |  | 0.000 |
| 16-24 | 708 (8.8) | 26 (2.6) | 682 (9.7) |  |
| 25-34 | 1129 (14.1) | 64 (6.3) | 1,065 (15.2) |  |
| 35-44 | 1337 (16.7) | 98 (9.7) | 1,239 (17.7) |  |
| 45-54 | 1407 (17.5) | 150 (14.8) | 1,257 (17.9) |  |
| 55-64 | 1266 (15.8) | 194 (19.2) | 1,072 (15.3) |  |
| 65-74 | 1226 (15.3) | 233 (23.0) | 993 (14.2) |  |
| 75+ | 949 (11.8) | 247 (24.4) | 702 (10.0) |  |
| **Ethnicity** |  |  |  | 0.000 |
| White | 7131 (88.9) | 973 (96.2) | 6,178 (87.9) |  |
| Non-White | 876 (10.9) | 37 (3.6) | 839 (12.0) |  |
| Missing | 15 (0.2) | 2 (0.2) | 13 (0.2) |  |
| **Household size** |  |  |  | 0.000 |
| 1 | 1,474 (18.4) | 249 (24.6) | 1,225 (18.5) |  |
| 2 | 3,145 (39.2) | 484 (47.8) | 2,661 (37.9) |  |
| 3 | 1,382 (17.2) | 130 (12.9) | 1,252 (17.9) |  |
| 4 | 1,347 (16.8) | 105 (10.4) | 1,242 (17.7) |  |
| 5 or more | 674 (8.4) | 44 (4.4) | 630 (9.0) |  |
| **Area level deprivation quintiles^a^** |  |  |  | 0.001 |
| Least deprived | 1719 (21.4) | 219 (21.6) | 1,500 (21.4) |  |
| Second lowest | 1641 (20.5) | 233 (23.0) | 1,408 (20.1) |  |
| Middle | 1656 (20.6) | 214 (21.2) | 1,442 (20.6) |  |
| Second highest | 1,446 (18.0) | 196 (19.4) | 1,250 (17.8) |  |
| Most deprived | 1596 (19.5) | 150 (14.8) | 1,410 (20.1) |  |
| **Well-being (WEMWBS score)** |  |  |  | 0.003 |
| Bottom 15^th^ centile | 981 (12.2) | 126 (12.5) | 855 (12.2) |  |
| Remainder | 5,088 (63.5) | 643 (63.5) | 4,445 (63.4) |  |
| Top 15^th^ centile | 973 (12.1) | 125 (12.4) | 848 (12.1) |  |
| Missing | 980 (12.3) | 118 (11.6) | 862 (12.3) |  |
| **Smoking status** |  |  |  | 0.000 |
| Never smoker | 3,982 (49.6) | 443 (43.8) | 3539 (50.5) |  |
| Ex-regular/Ex- occasional | 2,626 (32.7) | 414 (40.9) | 2212 (31.6) |  |
| Current | 1,367 (17.0) | 153 (15.1) | 1,214 (17.3) |  |
| Missing | 47 (0.6) | 2 (0.2) | 45(0.6) |  |
| **Alcohol consumption**^b^ |  |  |  | 0.000 |
| None | 3,252(40.5) | 372 (36.7) | 2,880 (41.1) |  |
| ≤2 units | 1,591 (19.8) | 251 (24.8) | 1340 (19.1) |  |
| >2 and ≤4 units | 1,043 (13.0) | 125 (12.4) | 918 (13.1) |  |
| > 4 and ≤8 units | 1,098 (13.7) | 151 (14.9) | 947 (13.5) |  |
| 8+ units | 1,030 (12.8) | 112 (11.1) | 918 (13.1) |  |
| Missing | 8 (0.1) | 1 (0.1) | 7 (0.1) |  |
| **BMI** ^c,d^ |  |  |  | 0.310 |
| Not overweight or obese | 2,788 (34.8) | 326 (32.2) | 2,462 (35.1) |  |
| Overweight | 2,899 (36.1) | 386 (38.1) | 2,513 (35.9) |  |
| Obese | 2,185 (27.2) | 280 (27.7) | 1,905 (27.2) |  |
| Missing | 150 (1.9) | 20 (2.0) | 130 (1.8) |  |
| **Physical activity (moderate) ^4^** |  |  |  | 0.004 |
| 1-2 days | 1,920 (23.9) | 204 (20.3) | 1,715 (24.5) |  |
| 3-4 days | 1,383 (17.2) | 164 (16.2) | 1,219 (17.4) |  |
| 5-6 days | 557(6.9) | 68 (6.7) | 489 (7.0) |  |
| 7 days | 1,146(14.0) | 175 (17.3) | 971 (13.9) |  |
| No days | 2,743 (14.3) | 355 (17.3) | 2,373 (33.9) |  |
| Refused | 276 (3.4) | 45 (4.5) | 243 (3.5) |  |
| **Clinical conditions** |  |  |  |  |
| Diabetes | 597 7.4) | 97 (9.6) | 500 (7.1) | 0.020 |
| Respiratory Disease | 582 (7.3) | 108 (10.7) | 474 (6.8) | 0.000 |
| Digestive disorders | 424 (5.3) | 90 (8.9) | 334 (4.8) | 0.000 |
| Genito-urinary disorders | 176 (2.2) | 37 (3.7) | 139 (2.0) | 0.001 |
| Mental Health condition | 502 (6.3) | 62 (6.1) | 440 (6.3) | 0.869 |
| Long-lasting illness | 4,602 (57.4) | 437 (43.2) | 4,165 (59.5) | 0.000 |

a Area level deprivation defined using Index of Multiple Deprivation 2015 scores

b Units of alcohol consumed on heaviest drinking day in past 7 days

c BMI was calculated using measured height and weight where available and supplemented with self-reported BMI among participants with no height and/or weight measurements (BMI categories: <25 kg/m^2^, not overweight or obese; 25 to <30 kg/m^2^, overweight; and 30 kg/m^2^ or more, obese).

d Underweight included with normal weight due to small numbers

e Number of days performing moderate physical activity in the past 7 days

^ χ2tests were used to determine the statistical significance of any difference in the distributions. Statistical significance level: p value <0.05

BMI, body mass index, WEMWBS, Warwick-Edinburgh Mental Well-being Scale

**Table S2** Factors associated with reporting HZ, stratified by gender (fully adjusted model)

|  | Female (N=4454) | | | | | Male (N=3568)) | | | |
| --- | --- | --- | --- | --- | --- | --- | --- | --- | --- |
|  | aOR | | 95% CI | | p-value^ | aOR | 95% CI | | p-value^ |
| Age-group |  |  | |  |  |  |  |  |  |
| 16-24 | ref |  | |  |  |  |  |  |  |
| 25-34 | 1.44 | 0.73 | | 2.83 | 0.291 | 1.22 | 0.50 | 2.97 | 0.666 |
| 35-44 | 1.91 | 1.03 | | 3.54 | 0.039 | 2.07 | 0.93 | 4.61 | 0.074 |
| 45-54 | 2.78 | 1.53 | | 5.05 | 0.001 | 2.91 | 1.26 | 6.72 | 0.012 |
| 55-64 | 4.46 | 2.41 | | 8.25 | 0.000 | 2.95 | 1.33 | 6.57 | 0.008 |
| 65-74 | 4.90 | 2.71 | | 8.88 | 0.000 | 5.78 | 2.50 | 13.35 | 0.000 |
| 75+ | 7.67 | 4.19 | | 14.03 | 0.000 | 8.02 | 3.42 | 18.82 | 0.000 |
| Ethnicity |  |  | |  |  |  |  |  |  |
| Non-White | ref |  | |  |  |  |  |  |  |
| White | 2.17 | 1.27 | | 3.69 | 0.004 | 1.72 | 0.98 | 3.03 | 0.059 |
| Well-being (WEMWBS score) |  |  | |  |  |  |  |  |  |
| Bottom 15^th^ centile | ref |  | |  |  |  |  |  |  |
| Remainder | 0.96 | 0.71 | | 1.31 | 0.801 | 0.92 | 0.61 | 1.41 | 0.712 |
| Top 15^th^ centile | 1.04 | 0.70 | | 1.55 | 0.836 | 0.92 | 0.55 | 1.52 | 0.735 |
| Household size |  |  | |  |  |  |  |  |  |
| 1 | ref |  | |  |  |  |  |  |  |
| 2 | 0.95 | 0.74 | | 1.21 | 0.671 | 1.20 | 0.84 | 1.72 | 0.320 |
| 3 | 1.13 | 0.78 | | 1.65 | 0.513 | 1.09 | 0.66 | 1.79 | 0.734 |
| 4 | 0.93 | 0.61 | | 1.43 | 0.740 | 1.53 | 0.93 | 2.52 | 0.092 |
| 5 or more | 0.85 | 0.48 | | 1.49 | 0.564 | 1.14 | 0.51 | 2.53 | 0.748 |
| BMI^a,b^ |  |  | |  |  |  |  |  |  |
| Not overweight or obese | ref |  | |  |  |  |  |  |  |
| Overweight | 1.01 | 0.81 | | 1.27 | 0.911 | 0.84 | 0.60 | 1.18 | 0.320 |
| Obese | 0.86 | 0.66 | | 1.13 | 0.280 | 0.86 | 0.60 | 1.24 | 0.431 |
| Area level Deprivation^c^ |  |  | |  |  |  |  |  |  |
| Least deprived | ref |  | |  |  |  |  |  |  |
| Second-lowest | 0.97 | 0.71 | | 1.33 | 0.853 | 1.40 | 0.93 | 2.11 | 0.106 |
| Middle | 1.05 | 0.78 | | 1.40 | 0.753 | 1.10 | 0.72 | 1.69 | 0.649 |
| Second-highest | 1.00 | 0.73 | | 1.35 | 0.975 | 1.34 | 0.87 | 2.07 | 0.187 |
| Most deprived | 0.92 | 0.65 | | 1.30 | 0.639 | 0.92 | 0.57 | 1.47 | 0.721 |
| Physical activity - moderate | |  | |  |  |  |  |  |  |
| No days | ref |  | |  |  |  |  |  |  |
| 1-2 days | 0.96 | 0.71 | | 1.29 | 0.791 | 0.74 | 0.52 | 1.05 | 0.094 |
| 3-4 days | 1.11 | 0.81 | | 1.52 | 0.520 | 1.14 | 0.76 | 1.73 | 0.521 |
| 5-6 days | 0.96 | 0.59 | | 1.56 | 0.870 | 1.28 | 0.81 | 2.01 | 0.294 |
| 7 days | 1.59 | 1.13 | | 2.24 | 0.007 | 1.03 | 0.69 | 1.54 | 0.871 |
| Smoking category |  |  | |  |  |  |  |  |  |
| Never smoker | ref |  | |  |  |  |  |  |  |
| Ex regular/Ex occasional smoker | 1.18 | 0.94 | | 1.48 | 0.145 | 0.99 | 0.74 | 1.31 | 0.930 |
| Current smoker | 1.47 | 1.09 | | 2.00 | 0.013 | 0.97 | 0.64 | 1.47 | 0.894 |
| Alcohol consumption^d^ |  |  | |  |  |  |  |  |  |
| None | ref |  | |  |  |  |  |  |  |
| ≤2 units | 1.10 | 0.85 | | 1.43 | 0.467 | 1.27 | 0.86 | 1.87 | 0.224 |
| >2 and ≤4 units | 0.93 | 0.66 | | 1.31 | 0.673 | 1.09 | 0.74 | 1.60 | 0.666 |
| > 4 and ≤8 units | 1.14 | 0.83 | | 1.58 | 0.415 | 1.25 | 0.82 | 1.89 | 0.297 |
| 8+ units | 1.04 | 0.68 | | 1.59 | 0.852 | 1.45 | 0.97 | 2.17 | 0.073 |
| Clinical conditions |  |  | |  |  |  |  |  |  |
| Diabetes^e^ | 1.03 | 0.70 | | 1.53 | 0.875 | 1.02 | 0.68 | 1.54 | 0.918 |
| Respiratory disease | 1.22 | 0.83 | | 1.78 | 0.307 | 1.44 | 0.89 | 2.35 | 0.139 |
| Digestive disorders | 1.70 | 1.18 | | 2.43 | 0.004 | 1.27 | 0.75 | 2.15 | 0.366 |
| Genito-urinary disorders | 0.96 | 0.51 | | 1.78 | 0.884 | 1.73 | 0.93 | 3.21 | 0.082 |
| Mental health condition | 0.92 | 0.60 | | 1.41 | 0.716 | 1.68 | 0.93 | 3.03 | 0.087 |

a BMI was calculated using measured height and weight where available and supplemented with self-reported BMI among participants with no height and/or weight measurements (BMI categories: <25 kg/m^2^, not overweight or obese; 25 to <30 kg/m^2^, overweight; and 30 kg/m^2^ or more, obese)

b Underweight included with normal weight due to small numbers

c Area level deprivation defined using Index of Multiple Deprivation 2015 scores

d Units of alcohol consumed on heaviest drinking day in past 7 days

e Doctor-diagnosed diabetes

^Statistical significance level: p value <0.05

BMI, body mass index, WEMWBS, Warwick-Edinburgh Mental Well-being Scale
